# Vertigo as a Brain Symptom

**Published:** 1875-03

**Authors:** 


Vertigo as a Brain Symptom.—Vertigo is considered an early indication of several cerebral affections, but it is well to remember that at a recent meeting of the Clinical Society of London (*Brit. Med. Jour.*, Oct. 31, 1874,) Mr. Carter and Dr. Hughlings Jackson adduce cases showing that this symptom, with others simulating brain-disease, may be caused by overstrained convergence of the eyes in short-sighted persons reading much without spectacles.—*Medical Record*, Feb. 6, 1875, p. 93.

